# Measures of wave intensity as a non-invasive surrogate for cardiac function predicts mortality in haemodialysis patients

**DOI:** 10.1093/ckj/sfae172

**Published:** 2024-06-27

**Authors:** Christopher C Mayer, Pantelis A Sarafidis, Julia Matschkal, Marieta Theodorakopoulou, Georg Lorenz, Artemios Karagiannidis, Susanne Angermann, Fotini Iatridi, Matthias C Braunisch, Antonios Karpetas, Marcus Baumann, Eva Pella, Uwe Heemann, Siegfried Wassertheurer, Christoph Schmaderer

**Affiliations:** AIT Austrian Institute of Technology, Center for Health & Bioresources, Medical Signal Analysis, Vienna, Austria; TU Wien, Institute for Analysis and Scientific Computing, Vienna, Austria; Aristotle University of Thessaloniki, Department of Nephrology, Hippokration Hospital, Thessaloniki, Greece; Technical University of Munich, School of Medicine, Klinikum rechts der Isar, Department of Nephrology, Munich, Germany; Aristotle University of Thessaloniki, Department of Nephrology, Hippokration Hospital, Thessaloniki, Greece; Technical University of Munich, School of Medicine, Klinikum rechts der Isar, Department of Nephrology, Munich, Germany; Aristotle University of Thessaloniki, Department of Nephrology, Hippokration Hospital, Thessaloniki, Greece; Technical University of Munich, School of Medicine, Klinikum rechts der Isar, Department of Nephrology, Munich, Germany; Aristotle University of Thessaloniki, Department of Nephrology, Hippokration Hospital, Thessaloniki, Greece; Technical University of Munich, School of Medicine, Klinikum rechts der Isar, Department of Nephrology, Munich, Germany; Therapeutiki Hemodialysis Unit, Thessaloniki, Greece; Technical University of Munich, School of Medicine, Klinikum rechts der Isar, Department of Nephrology, Munich, Germany; Aristotle University of Thessaloniki, Department of Nephrology, Hippokration Hospital, Thessaloniki, Greece; Technical University of Munich, School of Medicine, Klinikum rechts der Isar, Department of Nephrology, Munich, Germany; AIT Austrian Institute of Technology, Center for Health & Bioresources, Medical Signal Analysis, Vienna, Austria; TU Wien, Institute for Analysis and Scientific Computing, Vienna, Austria; Technical University of Munich, School of Medicine, Klinikum rechts der Isar, Department of Nephrology, Munich, Germany

**Keywords:** blood pressure, cardiac function, ESKD, haemodialysis, wave intensity analysis

## Abstract

**Background:**

Risk prediction in haemodialysis (HD) patients is challenging due to the impact of the dialysis regime on the patient's volume status and the complex interplay with cardiac function, comorbidities and hypertension. Cardiac function as a key predictor of cardiovascular (CV) mortality in HD patients is challenging to assess in daily routine. Thus the aim of this study was to investigate the association of a novel, non-invasive relative index of systolic function with mortality and to assess its interplay with volume removal.

**Methods:**

A total of 558 (373 male/185 female) HD patients with a median age of 66 years were included in this analysis. They underwent 24-hour ambulatory blood pressure monitoring, including wave intensity analysis [i.e. S:D ratio (SDR)]. All-cause and CV mortality served as endpoints and multivariate proportional hazards models were used for risk prediction. Intradialytic changes were analysed in tertiles according to ultrafiltration volume. During a follow-up of 37.8 months, 193 patients died (92 due to CV reasons).

**Results:**

The SDR was significantly associated with all-cause {univariate hazard ratio [HR] 1.36 [95% confidence interval (CI) 1.20–1.54], *P* < .001} and CV [univariate HR 1.41 (95% CI 1.20–1.67), *P* < .001] mortality. The associations remained significant in multivariate analysis accounting for possible confounders. Changes in the SDR from pre-/early- to post-dialytic averages were significantly different for the three ultrafiltration volume groups.

**Conclusion:**

This study provides well-powered evidence for the independent association of a novel index of systolic function with mortality. Furthermore, it revealed a significant association between intradialytic changes of the measure and intradialytic volume removal.

## Graphical Abstract

**Graphical Abstract:**
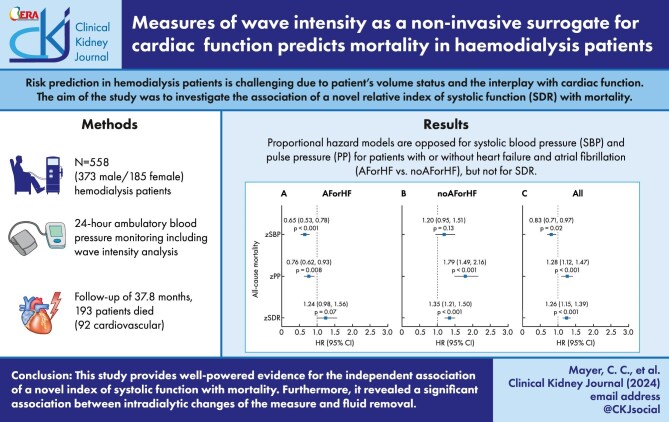

KEY LEARNING POINTS **What was known:**

- Risk prediction and blood pressure monitoring in haemodialysis (HD) patients are challenging.
- The dialysis regime impacts the patient's fluid status and there is a complex interplay of fluid balance with cardiac function, comorbidities and hypertension.

**This study adds:**

- The results of the study can play an important role in the management of hypertension and cardiovascular risk in HD patients, because it provides evidence that a novel non-invasive biomarker predicts mortality despite of the patient's cardiac status.
- There is a clear association between intradialytic changes of the biomarker and volume removal.

**Potential impact:**

- A potential impact of the current study is the fact that the index might work as a therapy target for intervention, as the study revealed a significant association between intradialytic changes in the index and fluid removal. Thus a modification might reduce mortality risk.

## INTRODUCTION

Morbidity and mortality rates are still unacceptably high in end-stage kidney disease (ESKD) patients undergoing haemodialysis (HD) [1, 2]. Heart failure (HF) and atrial fibrillation (AF) are common comorbidities in ESKD [3–6]. Their presence increases the risk of cardiovascular (CV) events. Further, HF and AF significantly modify the associations of blood pressure (BP) with adverse outcomes in these individuals [7]. Thus cardiac function may be impaired in dialysis patients and shall be considered in risk prediction, hypertension management and the definition of new therapy targets in ESKD.

Ambulatory BP monitoring (ABPM) is recommended for the diagnosis and treatment of hypertension in HD patients [8, 9] but has its limitations in risk prediction. The main limitation is the fact that the association of BP with mortality is non-linear and depends on cardiac function and subsequently on volume status [7, 10]. Evidence from the turn of the last century paved the way for pulse wave analysis (PWA) as an important tool for risk prediction in dialysis patients. This evidence was derived from relatively young dialysis patients with poorly managed hypertension and based on single office measurements [11–13].

In recent years, new, non-invasive PWA approaches based on wave intensity analysis were suggested to play an important role in risk prediction and intervention [14]. For selected measures, there is evidence for associations with CV events [15], cardiac mortality [16] and cognitive decline [17] in various patient cohorts. One promising measure is the so-called S:D ratio (SDR), which is seen as a novel, relative index of systolic function. It combines pressure and flow dynamics from start and end of cardiac contraction and is capable of capturing the ventricular function [18].

Thus the primary objective of this study was to investigate the association of this novel, relative index of systolic function from non-invasive wave intensity analysis with all-cause and CV mortality in a large cohort of HD patients with 24-hour ABPM and PWA. Furthermore, the association between intradialytic changes of the novel index and volume removal during HD was assessed.

## MATERIALS AND METHODS

### Study population

Data for the current study originate from the German ISAR study (ClinicalTrials.gov, NCT01152892) [19] and the Northern Greek Haemodialysis Network (NGHN) [20], which were prospective cohort studies evaluating CV outcomes in HD patients. The ISAR study was approved by the Ethics Committees of the Klinikum rechts der Isar of the Technical University Munich and the Bavarian State Board of Physicians and was designed as a prospective observational cohort study with the general aim of improving CV risk stratification in ESKD patients [19]. The NGHN study was approved by the Ethics Committee of the School of Medicine, Aristotle University of Thessaloniki and is a prospective observational cohort study with the main objective to explore patterns of BP and related parameters and their association with outcomes in HD patients [20]. The study adheres to the Declaration of Helsinki and all patients gave informed consent. Patients were included between September 2010 and January 2014 in Germany and between February 2013 and August 2017 in Greece.

Inclusion criteria for the combined study population were age ≥18 years, dialysis vintage ≥90 days, dialysis scheduled three times a week, willingness to perform ABPM including pulse wave analysis and provided written informed consent [19, 20]. Patients with ongoing infection, pregnancy, malignant disease with poor prognosis or a recent (<1 month) CV event were excluded from the study; details can be found elsewhere [19, 20].

**Figure 1:**
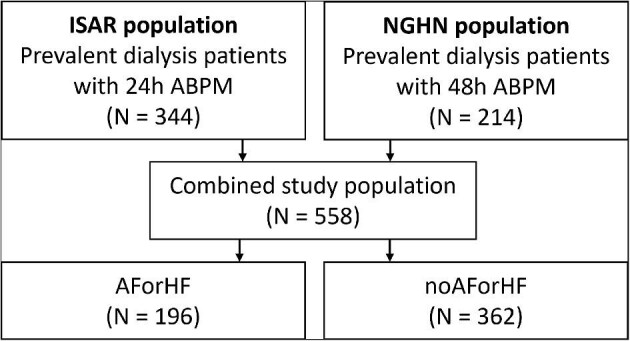
Flowchart of included patients.

For this analysis, 344 patients from the ISAR cohort [7] and 214 patients from the NGHN cohort were combined, leading to total study population of 558 patients (Fig. 1).

### Ambulatory measurements

The Mobil-O-Graph 24-hour PWA device (I.E.M., Stolberg, Germany) was used for ABPM including pulse wave analysis. It includes a validated brachial BP measurement unit [21, 22] and records brachial pulse waves at a diastolic level for 10 seconds [23]. The ABPM recordings including PWA were started before a midweek dialysis session and lasted for 24 hours (ISAR) or 48 hours (NGHN) using appropriate cuffs on the non-fistula arm; in the current study, only data from the first 24 hours were used. The devices were programmed to acquire BP measurements and pulse wave recording every 15 or 20 minutes during the day (8:00 a.m.–9:00 p.m. (ISAR) or 7:00 a.m.–11:00 p.m. (NGHN) and every 30 minutes at night (i.e. the remainder of the 24-hour interval).

BP data and the parameters from wave intensity analysis were averaged for 24 hours and intradialytic time period. Furthermore, pre-/early- and post-dialytic measures were derived as first three measurements in the first hour of the recording and the average of the measurements in the hour after the end of effective dialysis. Intradialytic averages were calculated as averages from 30 minutes after the recording started until the end of effective dialysis. Importantly, pulse wave and wave intensity analysis, which are based on 10-second pulse wave recordings from the brachial artery, include a quality indicator and discard single non-valid waveforms, e.g. affected by artefacts or arrhythmia due to AF [23, 24].

### Wave intensity analysis

A detailed description of the computational steps for pressure-only wave intensity analysis can be found in Supplementary Fig. S4 and Hametner *et al.* [18]. Briefly, brachial pulse waveforms obtained with the Mobil-O-Graph 24-hour PWA device were transformed to aortic pressure (*P*). Scaled aortic blood flow (*Q*) was determined by combining a Windkessel model relating pressure and flow with a minimal work criterion using the ARCSolver algorithms (AIT Austrian Institute of Technology, Vienna, Austria); the *Q* value was subsequently used as an estimate of flow velocity *U* [25]. Consequently, changes in pressure (Δ*P*) and flow velocity (Δ*U*) were computed and separated into forward and backward travelling components using the Waterhammer equations and a linearity assumption [18, 26]. Finally, forward and backward wave intensities are defined as the product of changes in pressure and flow velocity [18, 27]. Forward wave intensity is characterized by two dominant peaks, called S and D, which were shown to reflect systolic and late systolic/early diastolic ventricular function, respectively [28, 29]. Thus the SDR, which is independent of the scaling of flow velocity, combines pressure and flow dynamics from the start and end of cardiac contraction and is therefore suggested as a promising relative measure of systolic function [18].

### Data collection and laboratory measurements

In the ISAR and NGHN cohorts, patients’ clinical characteristics and regular medical treatment were assessed at the time of study inclusion. Blood samples and laboratory measurements were taken prior to a midweek dialysis session. Baseline comorbidities were determined in Germany as well in Greece by the treating physicians according to the patient's history. In the ISAR study, these were supported by the ISAR Endpoint Committee, including a cardiologist and a nephrologist [19]. Paroxysmal/permanent AF and HF were defined based on current treatment guidelines [30, 31] according to medical records and/or Holter electrocardiogram or echocardiography, respectively, as described elsewhere [7, 10].

### Endpoints

For this study, all-cause mortality served as the primary endpoint. CV mortality, defined as death due to sudden cardiac death, myocardial infarction, congestive HF or stroke or death after a CV procedure, was used as the secondary endpoint. The endpoints were adjudicated by the Endpoint Committee in Germany and by an independent investigator in Greece. The latest follow-up took place between April and September 2016 (ISAR) and in March 2019 (NGHN). Censoring was performed after kidney transplantation per transplantation date or the last day of dialysis when lost to follow-up or moved away.

### Statistical analysis

Continuous data are presented as mean [standard deviation (SD)] or median [interquartile range (IQR)] according to the Kolmogorov–Smirnov test. Categorical data are reported as total number (percentage). Patients were grouped according to the presence/absence of AF or HF due to the recent findings regarding the effects of cardiac status on the association of ambulatory systolic BP (SBP) or pulse pressure (PP) and dipping status with all-cause and CV mortality [7, 10]. Between-group differences were tested using the χ^2^ test for proportional data and Student's *t*-test or Mann–Whitney U-test, as appropriate, for continuous data. Interaction analysis was used to confirm the effects of cardiac status for ABPM in this cohort and to evaluate possible effects for the SDR. Univariate and multivariate Cox models were used for risk prediction. Assumptions of Cox models were checked using Schoenfeld residuals, examining influential observations and non-linearity by Martingale residuals. Cox models were adjusted for established risk factors in HD patients (i.e. model A: age, sex, diabetes mellitus and serum albumin; model B: model A plus ultrafiltration volume (UFV) and log-transformed dialysis vintage; model C: model B plus 24-hour SBP). Hazard ratios (HRs) are generally presented per unit increase; for visualization in Forest plots, normalized HRs (i.e. per SD increase) are used to allow better comparability. To assess the interrelation of the new index SDR with fluid removal during HD, patients were grouped in tertiles according to the UFV. Group comparisons of pre-/early- to post-dialytic changes were done by means of the Kruskal–Wallis test. Post hoc analysis was subsequently performed with Holm–Bonferroni correction. Furthermore, continuous association was tested using a linear regression model. Statistical significance was assumed at a 5% level. Statistical analysis was performed using Matlab R2014a and R2019b (MathWorks, Natick, MA, USA).

## RESULTS

### Patient characteristics

The study cohort consists of 558 patients (373 male/185 female) with a median age of 66 years (IQR 53–76). The prevalence of AF and/or HF was 35% at baseline, accounting for 196 patients in the AF and HF group. Of these, 146 (74%) had HF, 74 (38%) had AF and 37 (19%) both comorbidities. Furthermore, patients with HF were classified as HF with preserved ejection fraction (EF) [48 (33%)], HF with mid-range EF [60 (41%)], HF with reduced EF [30 (21%)] or unclassified, as detailed information was missing [8 (5%)]. Of the 74 patients with AF, 38 (51%) had paroxysmal and 36 (49%) had permanent AF. The median dialysis vintage of included patients was 35.5 months (IQR 17.3–70.5) with a diabetes prevalence of 35%. The presence of hypertension, defined as either high BP values in the ABPM or receiving antihypertensive medication at baseline, was noted in 522 (95%) patients. For detailed baseline characteristics, see Table 1 and Supplementary Table S1. For the group comparisons of pre-/early- to post-dialytic changes, a subset of patients (*n* = 438) was used, since for 120 patients either pre-/early- or post-dialytic averages were not available. Baseline characteristics for included and excluded patients can be found in Supplementary Table S2. Excluded patients were slightly older, had longer dialysis vintages and were slightly more often female. Included patients were more often on anticoagulation medication. The rest of the baseline characteristics, BP values and SDR were comparable between groups.

**Table 1:** Baseline characteristics.

Characteristics	AF or HF	No AF or HF	All
Patients, *n*	196	362	558
Age (years), median (IQR)	73.5 (64.5–79)	61 (49–72)	66 (53–76)
Male, *n* (%)	126 (64)	247 (68)	373 (67)
Body mass index (kg/m^2^), median (IQR)	25.5 (22.9–29.5)	25.3 (22.9–28.7)	25.4 (22.9–28.9)
Dialysis vintage (months), median (IQR)	32.4 (16.0–68.3)	37.1 (19.0–71.7)	35.5 (17.3–70.5)
Effective time of dialysis (h), median (IQR)	4.00 (4.00–4.26)	4.05 (4.00–4.42)	4.02 (4.00–4.38)
UFV (ml), mean (SD)	2168 (1068)	2152 (1093)	2158 (1083)
UF rate (ml/h), mean (SD)	522 (254)	506 (254)	511 (254)
Serum albumin (g/l), median (IQR)	39 (36–42)	40.8 (38–43)	40 (38–42.2)
Diabetes mellitus, *n* (%)	89 (45)	109 (30)	198 (35)
History of hypertension^a^, *n* (%)	181 (92)	341 (94)	522 (94)
Use of statin, *n* (%)	90 (46)	140 (39)	230 (41)
Use of anticoagulation meds, *n* (%)	89 (45)	94 (26)	183 (33)
Use of antihypertensive meds, *n* (%)	174 (89)	319 (88)	493 (88)
SBP (mmHg), mean (SD)	124 (18.6)	127 (17.3)	126 (17.8)
DBP (mmHg), mean (SD)	70.7 (11.2)	77 (12.2)	74.8 (12.2)
PP (mmHg), median (IQR)	52.1 (43–61.5)	47.8 (40.8–56.5)	49 (41.6–57.7)
Heart rate (bpm), mean (SD)	71.4 (9.61)	72.6 (9.7)	72.2 (9.67)
SDR (−), median (IQR)	2.49 (2.16–2.93)	2.47 (2.09–2.9)	2.48 (2.12–2.92)
All-cause mortality, *n* (%)	112 (57)	81 (22)	193 (35)
CV mortality, *n* (%)	48 (24)	44 (12)	92 (16)

^a^History of hypertension was defined as either the use of antihypertensive medication and/or 24-hour BP >140/90 mmHg.

Patients were followed for 37.8 months (IQR 25.0–57.8). Censoring of patients was done at kidney transplantation (*n* = 31) and at the date of moving away or loss to follow-up (*n* = 11). In total, 193 patients died (*n* = 112 in the AF and HF group); 92 due to CV causes (*n* = 48 in the AF and HF group). Causes of CV death can be found in Supplementary Table S3.

### All-cause and CV mortality

Interaction analysis confirmed the effects of cardiac function on the association of BP and PP with all-cause and CV mortality (*P* < .001 for SBP and PP for all-cause and CV mortality). As previously shown, the associations of SBP and PP with all-cause and CV mortality were J-/U-shaped in the study cohort. In contrast, the interaction term for the SDR was non-significant for all-cause (*P* = .65) and CV mortality (*P* = .93), thus underpinning a similar behaviour for both studied groups.

**Figure 2:**
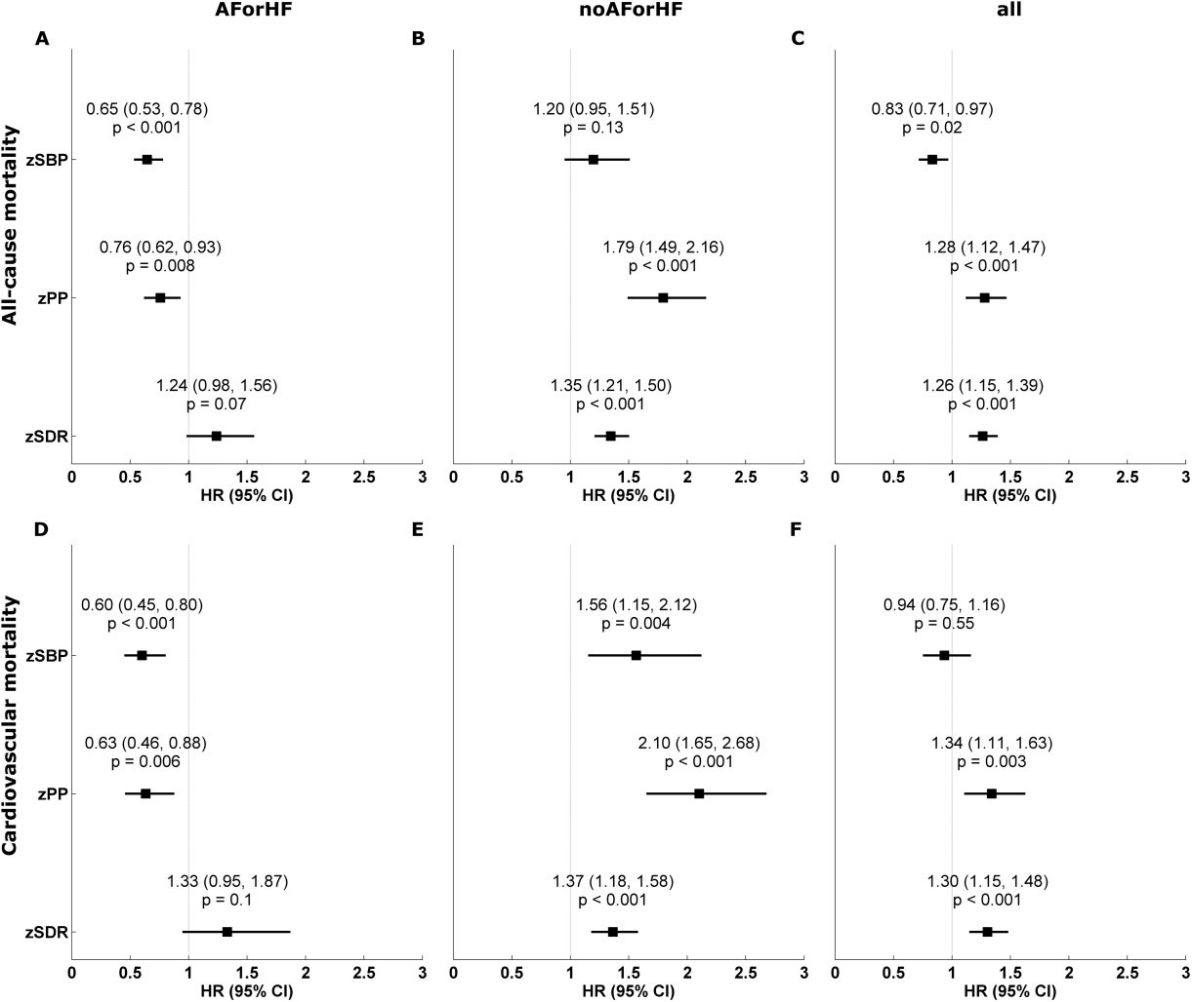
Association of normalized SBP, PP and SDR with mortality from univariate proportional Cox regression analysis. Univariate association of normalized SBP, PP and SDR (i.e. HR per SD) with **(A–C)** all-cause and **(D–F)** CV mortality for the AF or HF group (A, C), the No AF or HF group (B, E) and the whole study population (C, F). zSBP: z-score of SBP; zPP: z-score of PP; zSDR: z-score of SDR from wave intensity analysis.

In Tables 2 and 3, the results from linear Cox regression analysis are presented for the whole cohort and the study groups. SBP and PP were independent risk predictors for all-cause and CV mortality in the two groups after adjustment for possible confounders, with opposite associations according to cardiac function, except for SBP and all-cause mortality, where the association in the univariate analysis is of borderline significance [HR 1.010 (95% CI 0.997–1.023), *P* = .13]. The association between SBP and PP with the endpoints was inverse (i.e. negative; HR <1) in patients with HF and/or AF and positive (i.e. HR >1) in the second group. All associations were independent of other risk predictors. Furthermore, PP was not associated with outcome in patients with HF or HF when additionally adjusted for SBP. Proportional Cox regression analysis was not calculated for SBP and PP in the whole study cohort, as proportional assumptions were violated. Univariate HRs per SD are visualized in Forest plots in Fig. 2, highlighting the homogeneous results for the SDR (note: the results for SBP and PP in the whole cohort must be taken with caution due to the violation of the proportional assumption; Fig. 2C and F).

**Table 2:** Univariate and adjusted proportional HRs of all-cause mortality for BP, PP and SDR per unit increase.

	AF or HF (*n* = 196)		No AF or HF (*n* = 362)		All (*N* = 558)	
Model	HR (95% CI)	*P*-value	HR (95% CI)	*P*-value	HR (95% CI)	*P*-value
SBP	0.976 (0.965–0.986)	<.001	1.010 (0.997–1.023)	.13	–^e^	–^e^
SBP^b^	0.978 (0.967–0.989)	<.001	1.016 (1.004–1.028)	.01	–^e^	–^e^
SBP^c^	0.978 (0.967–0.990)	<.001	1.015 (1.003–1.027)	.02	–^e^	–^e^
SBP^d^	–^a^	–^a^	–^a^	–^a^	–^a^,^e^	–^a^,^e^
PP	0.978 (0.962–0.994)	.008	1.049 (1.033–1.065)	<.001	–^e^	–^e^
PP^b^	0.972 (0.955–0.990)	.002	1.032 (1.014–1.049)	<.001	–^e^	–^e^
PP^c^	0.973 (0.955–0.990)	.003	1.032 (1.015–1.050)	<.001	–^e^	–^e^
PP^d^	1.002 (0.970–1.034)	.92	1.045 (1.013–1.078)	.005	–^e^	–^e^
SDR	1.322 (0.978–1.787)	.07	1.475 (1.278–1.702)	<.001	1.356 (1.196–1.538)	<.001
SDR^b^	1.320 (0.974–1.789)	.07	1.413 (1.168–1.710)	<.001	1.264 (1.075–1.486)	.005
SDR^c^	1.361 (1.005–1.843)	.05	1.413 (1.165–1.714)	<.001	1.283 (1.096–1.503)	.002
SDR^d^	1.314 (0.972–1.777)	.08	1.388 (1.126–1.711)	.002	–^e^	–^e^

a Adjustment not performed since 24-hour SBP used as risk predictor. Adjustment for

b age, sex, diabetes mellitus and serum albumin (model A);

c model A plus UFV and log-transformed dialysis vintage (model B);

d model B plus 24-hour SBP (model C).

e Model and adjustment (model C) not calculated since proportional assumption violated for 24-hour SBP and PP (see Hametner and Wassertheurer [14]).

**Table 3:** Univariate and adjusted proportional HRs of CV mortality for BP, PP and SDR per unit increase.

	AF or HF (*n* = 196)		No AF or HF (*n* = 362)		All (*N* = 558)	
Model	HR (95% CI)	*P*-value	HR (95% CI)	*P*-value	HR (95% CI)	*P*-value
SBP	0.972 (0.956–0.988)	<.001	1.025 (1.008–1.043)	.004	–^e^	–^e^
SBP^b^	0.973 (0.956–0.991)	.003	1.030 (1.013–1.047)	<.001	–^e^	–^e^
SBP^c^	0.974 (0.956–0.991)	.004	1.030 (1.012–1.047)	<.001	–^e^	–^e^
SBP^d^	–^a^	–^a^	–^a^	–^a^	–^a^,^e^	–^a^,^e^
PP	0.964 (0.938–0.989)	.006	1.062 (1.042–1.083)	<.001	–^e^	–^e^
PP^b^	0.960 (0.933–0.988)	.005	1.056 (1.032–1.081)	<.001	–^e^	–^e^
PP^c^	0.960 (0.933–0.988)	.006	1.058 (1.033–1.082)	<.001	–^e^	–^e^
PP^d^	0.980 (0.932–1.031)	.44	1.068 (1.026–1.112)	.001	–^e^	–^e^
SDR	1.452 (0.932–2.262)	.1	1.501 (1.240–1.815)	<.001	1.413 (1.197–1.669)	<.001
SDR^b^	1.515 (0.965–2.377)	.07	1.459 (1.141–1.866)	.003	1.376 (1.116–1.698)	.003
SDR^c^	1.614 (1.021–2.550)	.04	1.461 (1.141–1.872)	.003	1.398 (1.140–1.715)	.001
SDR^d^	1.515 (0.958–2.395)	.08	1.452 (1.086–1.941)	.01	–^e^	–^e^

a Adjustment not performed since 24-hour SBP used as risk predictor. Adjustment for

b age, sex, diabetes mellitus and serum albumin (model A);

c model A plus UFV and log-transformed dialysis vintage (model B);

d model B plus 24-hour SBP (model C).

e Model C not calculated since proportional assumption violated for 24-hour SBP and PP (see Hametner and Wassertheurer [14]).

In contrast to the above, for the SDR, the situation was different and more homogeneous and, importantly, independent of the underlying presence or absence of AF or HF, as shown in Fig. 2. The results of the Cox regression models can again be found in Tables 2 and 3. Significant positive associations, or at least strong trends (i.e. borderline significant), were visible for the whole study cohort as well as in the two groups with HRs of similar magnitude. In the whole study cohort, the SDR was an independent risk predictor for all-cause mortality [univariate HR 1.356 (95% CI 1.196–1.538), *P* < .001; multivariable HR (model A) 1.264 (95% CI 1.075–1.486), *P* = .005; multivariable HR (model B) 1.283 (95% CI 1.096–1.503), *P* = .002]. Associations with CV mortality were similar [univariate HR 1.413 (95% CI 1.197–1.669), *P* < .001; multivariable HR (model A) 1.376 (95% CI 1.116–1.698), *P* = .003; multivariable HR (model B) 1.398 (95% CI 1.140–1.715), *P* = .001].

### Intradialytic analysis

Similar patterns for the association between intradialytic SDR and the primary and secondary outcomes in interaction and proportional Cox regression analyses could be seen (see Table 4). Associations and trends were again positive for the whole cohort and the two groups, whereas these were less pronounced for intradialytic values.

**Table 4:** Univariate and adjusted proportional HRs for intradialytic values for all-cause and CV mortality including 95% CIs for intradialytic SDR.

	AF or HF (*n* = 196)		No AF or HF (*n* = 362)		All (*N* = 558)	
Models	HR (95% CI)	*P*-value	HR (95% CI)	*P*-value	HR (95% CI)	*P*-value
All-cause mortality						
SBP	0.978 (0.967, 0.990)	<0.001	0.998 (0.985, 1.012)	0.8	–^e^	–^e^
SBP^b^	0.980 (0.968, 0.992)	0.001	1.006 (0.993, 1.019)	0.38	–^e^	–^e^
SBP^c^	0.981 (0.970, 0.993)	0.002	1.004 (0.991, 1.018)	0.51	–^e^	–^e^
SBP^d^	–^a^	–^a^	–^a^	–^a^	–^a^,^e^	–^a^,^e^
PP	0.987 (0.971, 1.002)	0.09	1.034 (1.018, 1.050)	<0.001	–^e^	–^e^
PP^b^	0.982 (0.966, 0.999)	0.03	1.017 (1.000, 1.035)	0.06	–^e^	–^e^
PP^c^	0.983 (0.967, 0.999)	0.04	1.017 (0.999, 1.035)	0.07	–^e^	–^e^
PP^d^	1.013 (0.984, 1.043)	0.39	1.032 (1.001, 1.062)	0.04	–^a^,^e^	–^a^,^e^
SDR	1.123 (0.885, 1.425)	0.34	1.095 (1.033, 1.161)	0.002	1.073 (1.016, 1.133)	0.01
SDR^b^	1.172 (0.921, 1.491)	0.2	1.123 (1.049, 1.201)	<0.001	1.106 (1.035, 1.181)	0.003
SDR^c^	1.198 (0.931, 1.543)	0.16	1.125 (1.051, 1.205)	<0.001	1.107 (1.038, 1.179)	0.002
SDR^d^	1.204 (0.939, 1.545)	0.14	1.129 (1.051, 1.212)	<0.001	–^e^	–^e^
CV Mortality						
SBP	0.977 (0.960, 0.995)	0.01	1.010 (0.992, 1.028)	0.29	–^e^	–^e^
SBP^b^	0.979 (0.961, 0.998)	0.03	1.017 (0.999, 1.036)	0.06	–^e^	–^e^
SBP^c^	0.979 (0.960, 0.998)	0.03	1.017 (0.998, 1.036)	0.08	–^e^	–^e^
SBP^d^	–^a^	–^a^	–^a^	–^a^	–^a^,^e^	–^a^,^e^
PP	0.982 (0.958, 1.007)	0.15	1.045 (1.024, 1.066)	<0.001	–^e^	–^e^
PP^b^	0.981 (0.956, 1.006)	0.13	1.034 (1.011, 1.058)	0.003	–^e^	–^e^
PP^c^	0.981 (0.956, 1.007)	0.14	1.035 (1.012, 1.059)	0.003	–^e^	–^e^
PP^d^	1.014 (0.970, 1.059)	0.55	1.049 (1.009, 1.092)	0.02	–^e^	–^e^
SDR	1.203 (0.851, 1.701)	0.3	1.119 (1.049, 1.193)	<0.001	1.100 (1.036, 1.167)	0.002
SDR^b^	1.261 (0.883, 1.800)	0.2	1.151 (1.071, 1.237)	<0.001	1.135 (1.062, 1.213)	<0.001
SDR^c^	1.349 (0.933, 1.950)	0.11	1.155 (1.073, 1.242)	<0.001	1.138 (1.067, 1.215)	<0.001
SDR^d^	1.330 (0.920, 1.924)	0.13	1.178 (1.087, 1.277)	<0.001	–^e^	–^e^

^a^Adjustment not performed since 24-hour SBP used as risk predictor. Adjustment for

^b^age, sex, diabetes mellitus and serum albumin (model A);

^c^model A plus UFV and log-transformed dialysis vintage (model B);

^d^model B plus 24-hour SBP (model C).

^e^Model C not calculated since proportional assumption violated for intradialytic SBP and PP.

**Figure 3:**
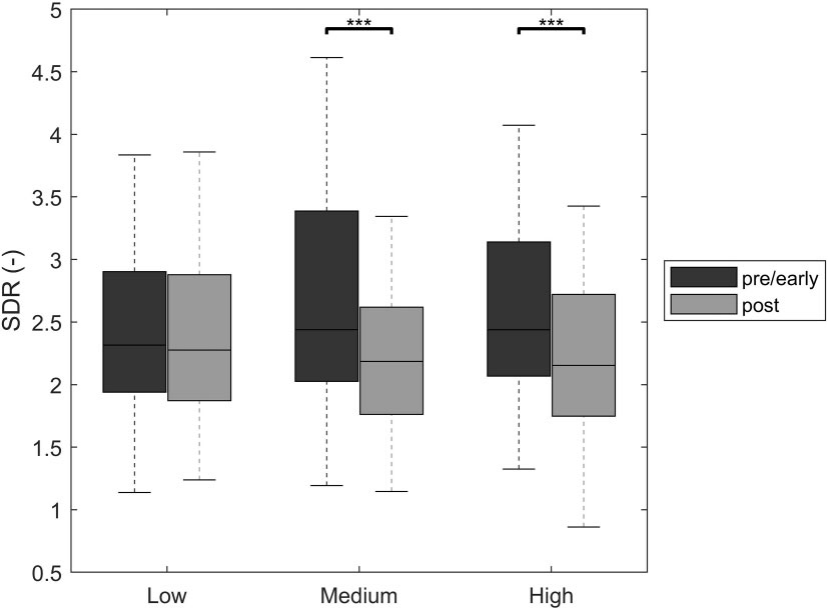
Changes from pre-/early- to post-dialytic SDR according to ultrafiltration tertiles. ***P < .001 from non-parametric, pairwise comparison with Holm–Bonferroni correction for multiple testing.

The mean UFV was 2158 ml (SD 1083) and tertiles of UFV were ≤1700 ml (*n* = 160; low), 1700–≤2600 ml (*n* = 140; medium) and >2600 ml (*n* = 138; high). The change from pre-/early- to post-dialytic SDR was −0.188 (IQR −0.751–0.278) in the whole cohort. Changes compared between UFVs were significantly different [low: −0.040 (IQR −0.459–0.422), medium: −0.252 (IQR −0.989–0.170); high: −0.347 (IQR −0.857–0.239); *P* < .001] (see Fig. 3). Post hoc analysis revealed significant differences between low versus medium and low versus high, respectively. The linear regression model for UFV and SDR confirmed this association (*P* = .005).

## DISCUSSION

The primary objective of this study was to investigate the association of a novel, relative index of systolic function from non-invasive wave intensity analysis with all-cause and CV mortality in a large cohort of HD patients with 24-hour ABPM and PWA. The main finding of this study is that the novel index SDR is an independent risk predictor for all-cause and CV mortality. Importantly, this association is independent of and not altered by the patients’ cardiac function represented by the presence or absence of AF and HF. To our knowledge, this is the first study to demonstrate that a parameter from non-invasive wave intensity analysis [18] is predictive for mortality in HD patients. Furthermore, the changes from pre-/early- to post-dialytic values highlight the association of total body volume and fluid removal during HD with CV function in these patients.

Results from previous work related to ABPM in the ISAR study (344 patients) showed a U-shaped association between 24-hour SBP or PP with adverse events; therein, the non-proportional association could be explained by the cardiac function [7]. These results could be confirmed in the combined dataset. As previously described [7], opposite linear associations (depending on the absence or presence of AF and HF) of 24-hour SBP and PP with the primary and secondary outcomes could be observed. This highlights the fact that not only BP management, including diagnosis and treatment, is challenging due to the complex interplay of cardiac function, fluid management and dialysis regime [8, 9], but also that risk prediction based on ambulatory BP can be significantly modified by the underlying cardiac function [7, 10].

The current study found that there is an independent association of the SDR, i.e. a novel parameter from wave intensity analysis, with all-cause and CV mortality in HD patients. Importantly, it overcomes the fact that risk prediction based on ambulatory BP depends on cardiac function, i.e. in contrast to SBP and PP, there is a positive association of the SDR with outcomes independent of the presence or absence of AF and/or HF. The SDR combines pressure and flow dynamics from the start and end of cardiac contraction, and thus is a promising relative measure of systolic function [18]. So far, there is limited evidence on the predictive power of parameters from wave intensity analysis, but our results are in line with previous findings. Manisty *et al.* [15] showed the independent association of wave intensity analysis measures with CV events in hypertensive patients and Vriz *et al.* [16] showed their association with CV mortality in patients with congestive heart failure and reduced EF based on ultrasound recordings at the carotid artery. Furthermore, there is evidence for an association of wave intensity measures with cognitive decline [17]. Of note, none of these results are based on pressure-only wave intensity analysis performed using a conventional occlusive cuff in an extended BP measurement on the brachial artery. The feasibility of pressure-only wave intensity analysis (i.e. deriving estimates of wave intensity based on the measurement of pressure only) was shown previously [32].

In clinical practice, risk prediction is important, but even more important is recognizing factors for intervention that may improve the prognosis. BP is an established modifiable factor in the general and several special populations, but in HD patients the situation is complex due to its non-proportional association with adverse events and the interference of cardiac status on this association. In contrast, (estimated) pulse wave velocity as a surrogate for arterial stiffness and a measure of vascular ageing is a strong and independent risk factor in HD patients [11, 33, 34] and other populations [35, 36], but it is much less suitable as a treatment target since it is affected by several factors and no relevant interventional evidence is available [37]. Results show that intradialytic changes in the SDR are associated with total body volume and fluid removal during HD. This might be a hint that the SDR is modifiable, which warrants further investigations. The SDR may be an alternative parameter for risk stratification, as the non-invasive approach using a standard occlusive cuff offers the opportunity to measure and monitor the parameter directly through the BP unit of the dialysis machine, thus allowing regular monitoring during the thrice-weekly dialysis sessions.

The strengths of the current study are the large sample size with an extensive follow-up and many fatal events. Inclusion criteria were defined very broadly to avoid any preselection of patients besides the mentioned exclusion criteria. With regards to limitations, analyses were limited to the on-dialysis day, since data from the ISAR study were recorded for 24 and not 48 hours. Furthermore, exact starting and end times of dialysis with respect to the ambulatory BP and PWA were not reported on an individual basis in the ISAR cohort, thus pre-/early- and post-dialytic averages could only be estimated on the information of dialysis length. Around 20% of all subjects were excluded from the pre-/early- to post-dialytic comparison due to missing values. These had clinical characteristics similar to those of included patients. Additionally, this study was limited by the absence of strain, strain rate, B-type natriuretic peptide (BNP) and tissue Doppler imaging for assessing systolic function or heart failure, and thus being able to compare these with the SDR. Associations of changes in the SDR with BNP as a marker for filling pressure could give interesting insights into heart failure patients, but the use of BNP in HD patients is limited due to the loss of its normal regulation and function in anephric patients. An additional uncontrolled factor is the possibility that SDR assessment is influenced by comorbidities, whereas the ARCSolver algorithms include quality controls and discard measurements affected by artifacts and arrythmias. This needs to be considered in the analysis and should be addressed in dedicated studies in the future. Furthermore, echocardiography was performed for all patients to determine HF, but unfortunately not at the time of PWA measurement; no Doppler measurements were performed within the study. Thus wave intensity analysis based on oscillometric measurements could not be validated in this cohort. The ARCSolver algorithms for pulse wave analysis and modelling flow have been validated extensively both non-invasively and invasively. Finally, included patients were from multiple centres in Munich, its suburban area and the area of northern Greece. Therefore, mainly Caucasians were included in the study and the results might not be generalizable to other ethnic groups.

In conclusion, this study provides well-powered evidence for the independent association of an index of systolic function from non-invasive analysis, which reflects systolic and late systolic/early diastolic ventricular function in the forward wave intensity, with mortality. Furthermore, it suggests that this measure might work as a therapy target for intervention. Further research is warranted.

## Supplementary Material

sfae172_Supplemental_File

## Data Availability

The datasets for this article are not publicly available because written informed consent did not include wording on data sharing. Reasonable requests to access data should be directed to the corresponding author.
