# Supplementary material for: Measures of wave intensity as a non-invasive surrogate for cardiac function predicts mortality in haemodialysis patients
Source: Clin Kidney J. 2024 Jun 27;17(7):sfae172. doi: 10.1093/ckj/sfae172 (PMC11270016; doi:10.1093/ckj/sfae172)
Supplement: sfae172_Supplemental_File [file sfae172_supplemental_file.pdf]

--- DATA SUPPLEMENT ---

Measure of wave intensity as non-invasive surrogate for cardiac function predicts mortality in hemodialysis patients

Christopher C. Mayer, PhD[1,2], Pantelis A. Sarafidis, PhD, MD[3], Julia Matschkal, MD[4], Marieta Theodorakopoulou, MD[3], Georg Lorenz, MD[4], Artemios Karagiannidis, MD[3], Susanne Angermann, MD[4], Fotini Iatridi, MD[3], Matthias C. Braunisch, MD[4], Antonios Karpetas, MD, PhD[5], Marcus Baumann, PhD, MD[4], Eva Pella, MD[3], Uwe Heemann, MD[4], Siegfried Wassertheurer, PhD[1,2], Christoph Schmaderer, MD[4]

[1] AIT Austrian Institute of Technology GmbH, Center for Health & Bioresources, Biomedical Systems, Giefinggasse 4, 1210 Vienna, Austria
[2] TU Wien, Institute for Analysis and Scientific Computing, Wiedner Hauptstr. 8-10, 1040 Vienna, Austria
[3] Aristotle University of Thessaloniki, Hippokration Hospital, Department of Nephrology, Konstantinoupoleos 49, 54642 Thessaloniki, Greece
[4] Technical University of Munich, School of Medicine, Klinikum rechts der Isar, Department of Nephrology, Ismaninger Strasse 22, 81675 Munich, Germany
[5] Therapeutiki Hemodialysis Unit, Thessaloniki, Greece

Table S1: Baseline characteristics of patients from the ISAR and the NGHN cohort separately.

Characteristic	NGHN	ISAR	All
N	214	344	558
Age (yr)	62.5 [52,74]	69 [55,77]	66 [53,76]
Sex-male, n (%)	139 (65 %)	234 (68 %)	373 (67 %)
Body mass index (kg/m²)	25.7 [23.2,29.5]	25.2 [22.8,28.7]	25.4 [22.9,28.9]
Dialysis vintage (mo)	28.0 [12.0,59.0]	41.1 [22.7,76.6]	35.5 [17.3,70.5]
Effective time of dialysis (h)	4.0 [4.0,4.0]	4.23 [4.0,4.5]	4.02 [4.0,4.38]
UFV (ml)	2057 (1003 SD)	2220 (1127 SD)	2158 (1083 SD)
UF rate (ml/h)	527 (263 SD)	501 (248 SD)	511 (254 SD)
Serum albumin (g/l)	40.2 (3.72 SD)	39.9 (4.14 SD)	40 (3.99 SD)
Presence of diabetes, n (%)	63 (29 %)	135 (39 %)	198 (35 %)
History of hypertension*, n (%)	196 (92 %)	326 (95 %)	522 (94 %)
Use of statin, n (%)	94 (44 %)	136 (40 %)	230 (41 %)
Use of anticoagulation med, n (%)	131 (61 %)	52 (15 %)	183 (33 %)
Use of antihypertensive med, n (%)	179 (84 %)	314 (91 %)	493 (88 %)
SBP (mmHg)	130 (18.1 SD)	123 (17 SD)	126 (17.8 SD)
DBP (mmHg)	78.5 (12 SD)	72.4 (11.8 SD)	74.8 (12.2 SD)
PP (mmHg)	50.6 [42.8,60]	48.7 [41.2,56.5]	49 [41.6,57.7]
Heart rate (1/min)	73.2 (8.99 SD)	71.5 (10 SD)	72.2 (9.67 SD)
SDR (-)	2.43 [2.07,2.77]	2.51 [2.16,3.01]	2.48 [2.12,2.92]
All-cause mortality, n (%)	78 (36 %)	115 (33 %)	193 (35 %)
CV mortality, n (%)	45 (21 %)	47 (14 %)	92 (16 %)

Results are presented as mean (standard deviation) and median [inter-quartile range] for normally and non-normally distributed data, respectively; categorical data as total number (percentage).

Abbreviations: UF, ultrafiltration; UFV, ultrafiltration volume; SBP, systolic blood pressure; DBP, diastolic blood pressure; PP, pulse pressure; SDR, S to D ratio from wave intensity analysis; AF, atrial fibrillation; HF, heart failure; CV, cardiovascular. *History of hypertension was defined as either use of antihypertensive medication and/or 24h blood pressure >140/90 mmHg.

Table S2: Baseline characteristics of excluded and included patients of pre-/early- and post-dialytic analysis.

Characteristic	Pre-/Early- and /Post-dialytic Subgroup	Excluded	All
N	438	120	558
Age (yr)	65 [52,75]	69 [58,77]	66 [53,76]
Sex-male, n (%)	282 (64 %)	91 (76 %)	373 (67 %)
Body mass index (kg/m²)	25.5 [23,29.3]	25 [22.6,28.1]	25.4 [22.9,28.9]
Dialysis vintage (mo)	33.4 [16.4,67]	47.2 [23.7,79.6]	35.5 [17.3,70.5]
Effective time of dialysis (h)	4.00 [4.00,4.37]	4.17 [4.00,4.43]	4.02 [4.00,4.38]
UFV (ml)	2151 (1088 SD)	2180 (1070 SD)	2158 (1083 SD)
UF rate (ml/h)	512 (256 SD)	510 (248 SD)	511 (254 SD)
Serum albumin (g/l)	40.0 [38.0,42.3]	39.6 [37.9,42.0]	40.0 [38.0,42.2]
Presence of diabetes, n (%)	159 (36 %)	39 (33 %)	198 (35 %)
History of hypertension*, n (%)	410 (94 %)	112 (93 %)	522 (94 %)
Use of statin, n (%)	184 (42 %)	46 (38 %)	230 (41 %)
Use of anticoagulation med, n (%)	155 (35 %)	28 (23 %)	183 (33 %)
Use of antihypertensive med, n (%)	388 (89 %)	105 (88 %)	493 (88 %)
SBP (mmHg)	132 [119,145]	129 [114,146]	132 [119,145]
DBP (mmHg)	80.8 (13.9 SD)	80.7 (15.6 SD)	80.8 (14 SD)
PP (mmHg)	50.5 [42.4,60.8]	50.8 [43.5,63.1]	50.5 [42.5,60.8]
Heart rate (1/min)	71.7 (10.6 SD)	74.7 (13.6 SD)	71.9 (10.9 SD)
SDR (-)	2.48 [2.12,2.88]	2.46 [2.11,3.04]	2.48 [2.12,2.92]
All-cause mortality, n (%)	150 (34 %)	43 (36 %)	193 (35 %)
CV mortality, n (%)	71 (16 %)	21 (18 %)	92 (16 %)

Results are presented as mean (standard deviation) and median [inter-quartile range] for normally and non-normally distributed data, respectively; categorical data as total number (percentage).

Abbreviations: UF, ultrafiltration; UFV, ultrafiltration volume; SBP, systolic blood pressure; DBP, diastolic blood pressure; PP, pulse pressure; SDR, S to D ratio from wave intensity analysis; AF, atrial fibrillation; HF, heart failure; CV, cardiovascular. *History of hypertension was defined as either use of antihypertensive medication and/or 24h blood pressure >140/90 mmHg.

Table S3: Reasons for cardiovascular death

In the table, the reasons for cardiovascular death are presented for the whole study population and the two dedicated groups based on atrial fibrillation and heart failure.

Reasons	AForHF (N=196)	noAForHF (N=362)	All (N=558)
Sudden cardiac death	31	23	54
Myocardial infarction	3	6	9
Heart failure	7	4	11
Major stroke	3	3	6
Cardiac surgical procedure	0	2	2
Pulmonary embolism	1	1	2
Other cardiovascular reasons	3	5	8
Number of cardiovascular deaths	48	44	92

Abbreviations: AF, atrial fibrillation; HF, heart failure.

Figure S4: Detailed description and visualization for calculation of SDR (updated and based on own prior work [1]): Brachial pulse waveforms were obtained with the oscillometric Mobil-O-Graph 24-hour PWA device (A) and transformed to the aortic pressure P using the validated ARCSolver Transfer Function (B). Aortic blood flow Q was then determined by combining a Windkessel model relating pressure and flow with a minimal work criterion using the ARCSolver® algorithms (AIT Austrian Institute of Technology GmbH, Vienna, Austria) as described in [2] (C). Aortic blood flow was subsequently used as an estimate of flow velocity U [3]. Since PU-loop during early systole is approximately linear, pulse wave velocity c times blood density ρ, which reflects the blood density (1050 kg/m^3) is estimated from the slope (D). Consequently, changes in pressure (dP) and flow velocity (dU) were computed and separated into forward and backward travelling components using the Waterhammer equations $dP_{f,b} = \pm\rho c\ dU_{f,b}$ and a linearity assumption $dP = dP_f + dP_b$ and $dU = dU_f + dU_b$ [1,4], where dP and dQ denote changes per time step and subscripts f and b denote forward and backward travelling components, respectively. Finally, forward and backward wave intensities are defined as the product of changes in pressure and flow velocity as $dI_{f,b} = dP_{f,b} * dU_{f,b}$, see E [1,5]. Forward wave intensity is characterized by two dominant peaks, called S and D.

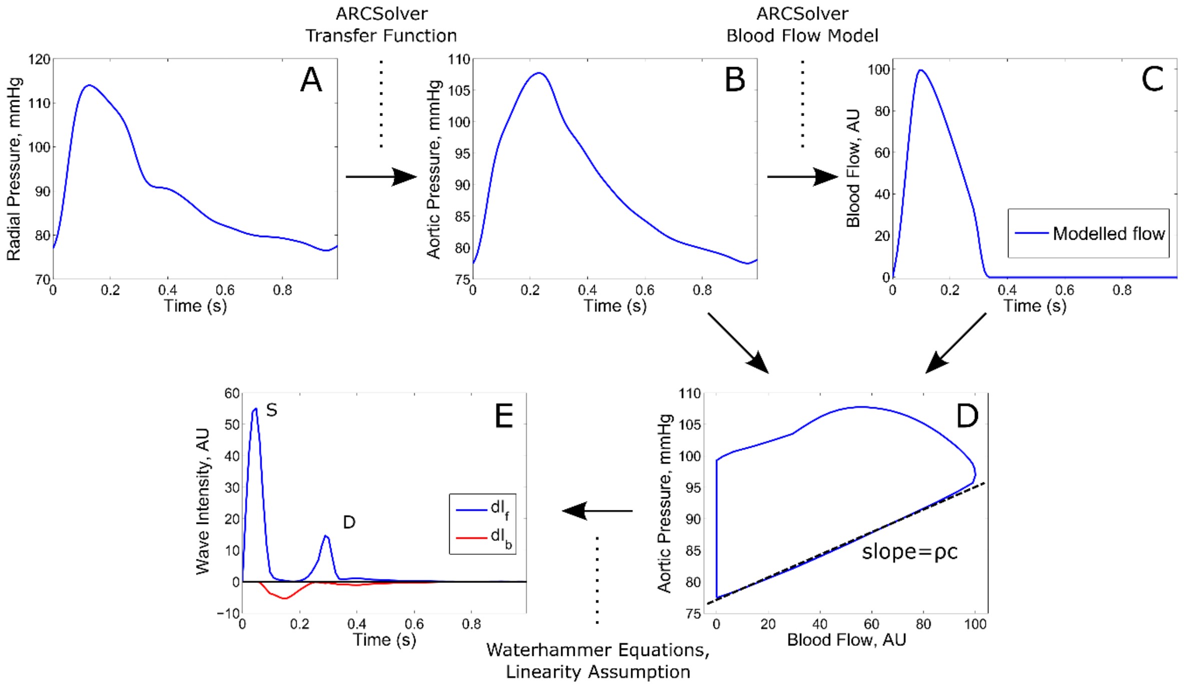

[1] Hametner B, Parragh S, Weber T, Wassertheurer S. Wave intensity of aortic root pressure as diagnostic marker of left ventricular systolic dysfunction. PLoS One. 2017;12(6):e0179938. doi: 10.1371/journal.pone.0179938.

[2] Hametner B, Wassertheurer S, Kropf J, Mayer C, Holzinger A, Eber B, Weber T. Wave reflection quantification based on pressure waveforms alone--methods, comparison, and clinical covariates. Comput Methods Programs Biomed. 2013;109(3):250-9. doi: 10.1016/j.cmpb.2012.10.005.

[3] Hametner B, Wassertheurer S, Kropf J, Mayer C, Holzinger A, Eber B, et al. Wave reflection quantification based on pressure waveforms alone-Methods, comparison, and clinical covariates. Comput Meth Programs Biomed 2013; 109(3):250–9.

[4] Parker K, Jones C. Forward and backward running waves in the arteries: analysis using the method of characteristics. J Biomech Eng 1990; 112(3):322–6.

[5] Mynard JP, Kondiboyina A, Kowalski R, Cheung MMH, Smolich JJ. Measurement, Analysis and Interpretation of Pressure/Flow Waves in Blood Vessels. Front Physiol. 2020 Aug 27;11:1085. doi: 10.3389/fphys.2020.01085.
